# Identifying an effective Chinese herbal medicine for reducing postoperative hidden bleeding and stabilizing blood volume following intertrochanteric femur fracture: a Bayesian network meta-analysis of randomized controlled trials

**DOI:** 10.1186/s13018-024-05379-3

**Published:** 2024-12-26

**Authors:** Xiaowei Wu, Linglan Yan, Tianran Wu, Shaochen Tu, Yanbin Lin

**Affiliations:** 1 Department of Orthopaedics, Fuzhou Second General Hospital, Fujian Medical University, Fuzhou, 350007 China; 2 Quanzhou Orthopedic-Traumatological Hospital, Quanzhou, 362000 China

**Keywords:** Intertrochanteric femur fracture, Chinese herbal medicine, Hidden bleeding, Network meta-analysis

## Abstract

**Objective:**

Chinese herbal medicine (CHM) is extensively utilized in managing hidden bleeding following an intertrochanteric femur fracture (IFF). Despite its widespread use, the most effective CHM approach for addressing hidden bleeding post-IFF remains unclear. A Bayesian network meta-analysis was conducted to identify a CHM that both effectively reduces hidden bleeding and stabilizes blood volume after IFF.

**Methods:**

Comprehensive electronic searches were performed on databases in both Chinese and English, including the Chinese National Knowledge Infrastructure, Wanfang Data Knowledge Service Platform (Wanfang), China Science and Technology Journal Database (VIP), PubMed, Web of Science, and Cochrane Library. All randomized controlled trials (RCTs) employing CHM to address hidden bleeding post-IFF, up to December 31, 2023, were thoroughly reviewed. The analysis utilized Stata17.0 and Review Manager 5.4 software, tailored to the frequentist framework.

**Results:**

Thirty-one articles were included, encompassing 17 interventions and 2076 patients. The systematic analysis revealed that: For improving postoperative haemoglobin (HB), the top three interventions were conventional treatment combined with Yiqi Buxue decoction, Yangxue Rougan decoction, and Sanqi powder; For reducing hidden bleeding post-surgery, the leading interventions were conventional treatment combined with Siwu decoction, Yangxue Rougan decoction, and Qitian Keli prescription; For enhancing the Harris score, the top interventions were conventional treatment combined with Bazhen decoction, Danggui Buxue decoction, and Sanqi powder; Descriptive analysis indicated minimal adverse reactions and enhanced safety overall.

**Conclusion:**

CHM presents a viable method for treating hidden blood loss after IFF. Yangxue Rougan decoction notably excels in reducing hidden bleeding and stabilizing HB levels, making it a preferred option. Nevertheless, further rigorous RCT studies are essential to compare various CHM treatments to establish the most effective options for clinicians.

*PROSPERO registration number* CRD42023489292.

**Supplementary Information:**

The online version contains supplementary material available at 10.1186/s13018-024-05379-3.

## Introduction

**Fig. 1:**
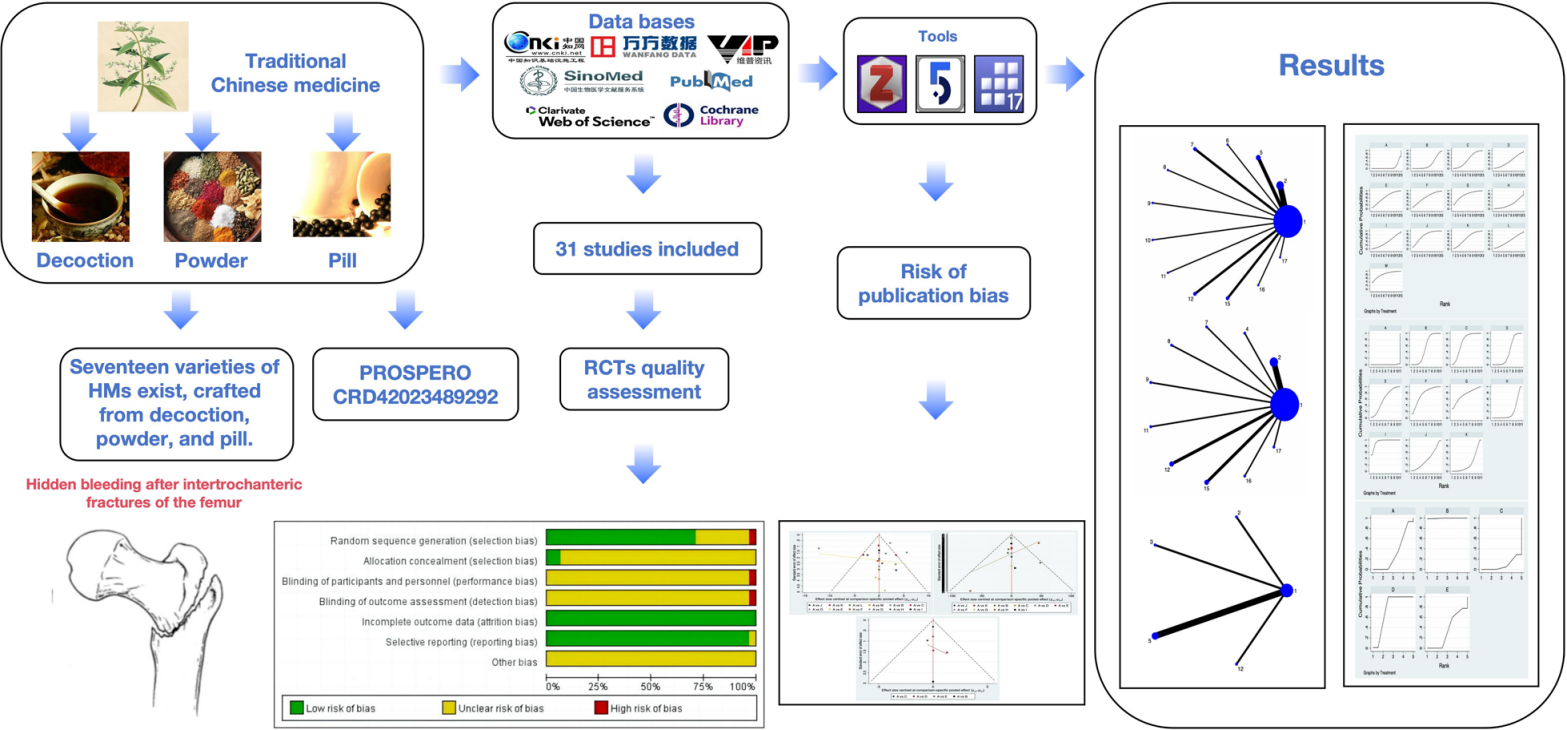
Experimental flowchart of this Bayesian network Meta-analysis

IFF is a prevalent orthopaedic condition [1], defined as a fracture occurring above the lesser trochanter and below the base of the femoral neck, constituting 3–4% of all human fractures and 50–60% of hip fractures [2]. As the population ages, the incidence of IFF in the elderly has significantly increased, along with the associated mortality rate [3]. Prompt internal fixation surgery is the most effective method to restore hip function in elderly patients, facilitating early mobilization and functional exercises to prevent or manage complications arising from prolonged bed rest [4–6]. Hidden blood loss refers to the discrepancy between the observed postoperative haemoglobin (HB) decline and the amount of intraoperative visible blood loss. The blood loss caused by joint space, intra-articular hemorrhage, hemolysis, and tissue space is ignored, and this part is the hidden blood loss [7]. Research indicates that perioperative blood loss during hip internal fixation can exceed 800 ml, with hidden blood loss being a significant factor [8]. Therefore, it is necessary to take effective measures to prevent postoperative hidden bleeding. Quaranta M et al. discovered that experienced orthopaedic surgeons can minimize blood loss and reduce anaemia risk in elderly hip fracture cases; however, the availability of such expertise is limited, and not all patients can access it [9]. Current treatments primarily include blood transfusions, iron supplementation, antifibrinolytic drugs, and functional exercises [10]. While autologous and allogeneic blood transfusions can rapidly elevate HB levels, they do not address the underlying issue of hidden blood loss [11]. Iron supplements support HB synthesis and red blood cell maturation but do not prevent hidden blood loss [12]. Early postoperative rehabilitation exercises enhance lower limb circulation and muscle strength and promote blood infiltration and absorption, albeit with limited efficacy [13]. Anti-fibrinolytic drugs, such as tranexamic acid, primarily function through an anti-fibrinolytic enzyme. This enzyme strongly adsorbs to the lysine-binding sites on plasmin and plasminogen, inhibiting their binding to fibrin. This inhibition significantly prevents the decomposition of fibrin caused by plasmin. Moreover, in the presence of α2-macroglobulin and other anti-fibrinolytic enzymes in serum, the anti-fibrinolytic effect of these drugs becomes more pronounced, enhancing their hemostatic efficacy [14]. However, tranexamic acid has a risk of deep vein thrombosis [15], and the hemostatic effect of tranexamic acid alone is not significant. Many studies have shown that the combination of CHM and tranexamic acid has better efficacy. Following complex processing and compatibility with various drugs, CHM is characterized by minimal side effects and consistent therapeutic efficacy [16]. The mechanism of action of CHM is well-documented, with pharmacological studies identifying components such as alkaloids, phenylpropanoids, quinones, terpenes, flavonoids, organic acids, glycosides, and steroids that exhibit hemostatic activity [17]. Thus, it is essential to explore effective treatment methods within the rich repository of CHM. Currently, multiple CHMs are available for treating postoperative hidden bleeding in IFF. Despite direct evidence indicating the superiority of certain CHMs over chemical drugs or placebos in managing IFF postoperative hidden bleeding [18], the optimal regimen among various CHMs remains uncertain and contentious. Previous studies reveal two primary outcomes: (1) CHM treatment reduces hidden blood loss in IFFs, with HB levels nearing normal [19, 20]; (2) CHM treatment decreases hidden blood loss, but HB levels remain significantly below normal [21, 22]. This discrepancy might be related to CHM's blood-producing capabilities. Some studies focused solely on postoperative hidden blood loss [23, 24], while others examined only HB values [25–27]. A few studies included both indicators but did not compare them comprehensively [20, 28, 29], and those that did often suffered from low-quality evidence and investigated only a single CHM [30]. Identifying a CHM that effectively reduces blood loss and stabilizes HB levels among various options will significantly aid clinicians in mitigating blood loss and reducing mortality in IFFs (Fig. 1).

## Methods

### General guidelines of the study

This study was conducted following the Cochrane Criteria and the PRISMA extension statement for reporting systematic reviews with network meta-analyses of healthcare interventions [31]. The study protocol is registered in the International Prospective Register of Systematic Reviews (PROSPERO) under the registration code CRD42023489292.

### Information sources and search strategy

Randomized controlled trials (RCTs) focusing on the clinical treatment of hidden bleeding in postoperative IFF were sourced from databases like CNKI, Wanfang, VIP, PubMed, Cochrane Library, and Web of Science. From the start of building the database until December 31, 2023, searches were conducted using Medical Subject Headings and free words. The Chinese search terms included the names of femoral intertrochanteric fracture, femoral intertrochanteric fracture, hidden hemorrhage, hidden blood loss, soup, formula, pill, powder, and Chinese medicine and included Chinese medicine prescriptions. The Chinese database takes CNKI as an example, the search formula is SU = (' intertrochanteric fracture of femur ' + ' intertrochanteric fracture of femur ') AND (' hidden bleeding ' + ' hidden blood loss ') AND (' decoction ' + ' prescription ' + ' pill ' + ' powder ' + ' traditional Chinese medicine ') AND (' random ' + ' randomized control ' + ' RCT '), After advanced search and manual screening, eighty-four articles were obtained. The English search Medical Subject Headings include Hip Fractures, Hemorrhage, Herbal Medicine, Randomized Controlled Trial[Publication Type] and their corresponding free words. Taking PubMed as an example, the search strategy of the English database is as follows:(((Randomized Controlled Trial[Publication Type]) AND (Hemorrhage OR Hemorrhages OR Bleeding)) AND (Hip Fractures OR Fractures, Hip OR Intertrochanteric Fractures OR Fractures, Intertrochanteric OR Trochanteric Fractures OR Fractures, Trochanteric OR Trochlear Fractures, Femur OR Femur Trochlear Fracture OR Femur Trochlear Fractures OR Fracture, Femur Trochlear OR Fractures, Femur Trochlear OR Trochlear Fracture, Femur OR Femoral Trochlear Fractures OR Femoral Trochlear Fracture OR Fracture, Femoral Trochlear OR Fractures, Femoral Trochlear OR Trochlear Fracture, Femoral OR Trochlear Fractures, Femoral OR Subtrochanteric Fractures OR Fractures, Subtrochanteric)) AND (("Herbal Medicine"[Mesh] OR Herbal Medicine OR Medicine, Herbal OR Hawaiian Herbal Medicine OR Herbal Medicine, Hawaiian OR Medicine, Hawaiian Herbal OR La'au Lapa'au OR Laau Lapaau OR La au Lapa au OR Herbalism). According to the PubMed retrieval strategy, two articles were retrieved. (The complete search formula is detailed in the supplementary file).

### Eligibility criteria

#### Inclusion criteria

1. Eligibility required RCTs to meet specific criteria for participants, interventions, controls, and outcome metrics, with publications in either English or Chinese.
2. Patients diagnosed with IFF based on diagnostic criteria established by authoritative organizations were included, regardless of age, race, gender, or disease course [32].
3. The experimental group received oral CHM combined with conventional treatment, whereas the control group received only conventional treatment. Oral CHM interventions could be in the form of decoctions, powders, or pills, provided their composition was clear.
4. RCTs were required to report the following main indicators: (1) Postoperative HB; (2) Postoperative hidden blood loss (calculated by the Gross formula) [33]; (3) Postoperative Harris score [34]. The frequency of adverse reactions following surgery was a secondary measure. These primary and secondary metrics are derived from the latest expert recommendations on IFF clinical studies.

#### Exclusion criteria

1. Exclusions included systematic reviews, conference summaries, clinical advisories, and research protocols.
2. The following documents were excluded: (1) Repeated publications; (2) Incomplete data or errors; (3) Studies with unexcluded comorbidities; (4) Studies lacking outcome indicators relevant to this research; (5) Studies where the treatment duration differed between experimental and control groups.
3. The intervention was only oral CHM, and external CHM was excluded.

### Choosing studies, gathering data, evaluating bias risks, and assessing the quality of evidence

Based on the stated criteria for inclusion and exclusion, two researchers independently selected the studies, and in case of discrepancies during the verification phase, a third researcher adjudicated to reach a consensus. The retrieved literature was imported into Zotero software for initial screening, with titles and abstracts reviewed to exclude studies not meeting inclusion standards. The remaining literature was then thoroughly reviewed, and those meeting the criteria were included. Information such as the author's name, case count in each category, mean age, gender, intervention strategies, treatment mode, and outcome metrics were compiled into an Excel spreadsheet for further analysis.

Two independent assessors evaluated the quality of the included literature. The Review Manager 5.4 software was used to assess risk bias, which was subsequently verified. The Cochrane tools' quality evaluation criteria included: (I) selection bias from random sequence generation and concealment; (II) performance bias from blinding of participants and personnel; (III) detection bias from blinding of outcome assessment; (IV) attrition bias from incomplete outcome data; (V) reporting bias from selective reporting; and (VI) other biases. Each criterion was categorized into high risk, uncertain risk, and low risk [35]. The GRADE system was used to evaluate the quality of evidence [36]. The GRADE system classifies the quality of evidence into four categories: high, medium, low, and very low. Based on the results of Meta-analysis, the GRADE evidence quality grading system was used to evaluate the evidence quality level of the outcome indicators through 5 downgrading conditions and 3 upgrading conditions. The five conditions for downgrading were risk of bias, inconsistency, indirectness, inaccuracy and publication bias. For any one of the five conditions, the quality of evidence can be downgraded by one level (serious) or two levels (very serious) depending on the severity of the problem. The quality of evidence can be downgraded to very low at best.

### Statistical analysis

The magnitude of statistical effects on continuous variables was quantified using mean difference (MD) or standardized mean difference (SMD) with 95% confidence intervals (CI). Traditional meta-analyses were conducted using RevMan 5.4 software. Heterogeneity among studies was assessed using the I^2^ statistic. If the heterogeneity test was not statistically significant (I^2^ < 50%), the fixed effect model was used, and the random effect model was used if the heterogeneity test was statistically significant (I^2^ > 50%). When the heterogeneity among studies was large, subgroup analysis was used according to the categorie of CHM to explore the source of heterogeneity. If the heterogeneity between studies could not be reduced, only descriptive analysis was employed. Based on the frequency framework, mvmeta and network packages of Stata 17.0 software were used for network meta-analysis, and evidence network maps were drawn to visualize relationships between interventions. The surface under the cumulative ranking curve (SUCRA) was calculated to compare the efficacy of interventions for each outcome index, and a "comparison-correction" funnel plot was used to assess potential small sample effects or publication bias. The inconsistency test was primarily used to assess the concordance level between direct and indirect comparative outcomes, required when there was a closed loop.

## Results

### Study selection and study characteristics

**Fig. 2:**
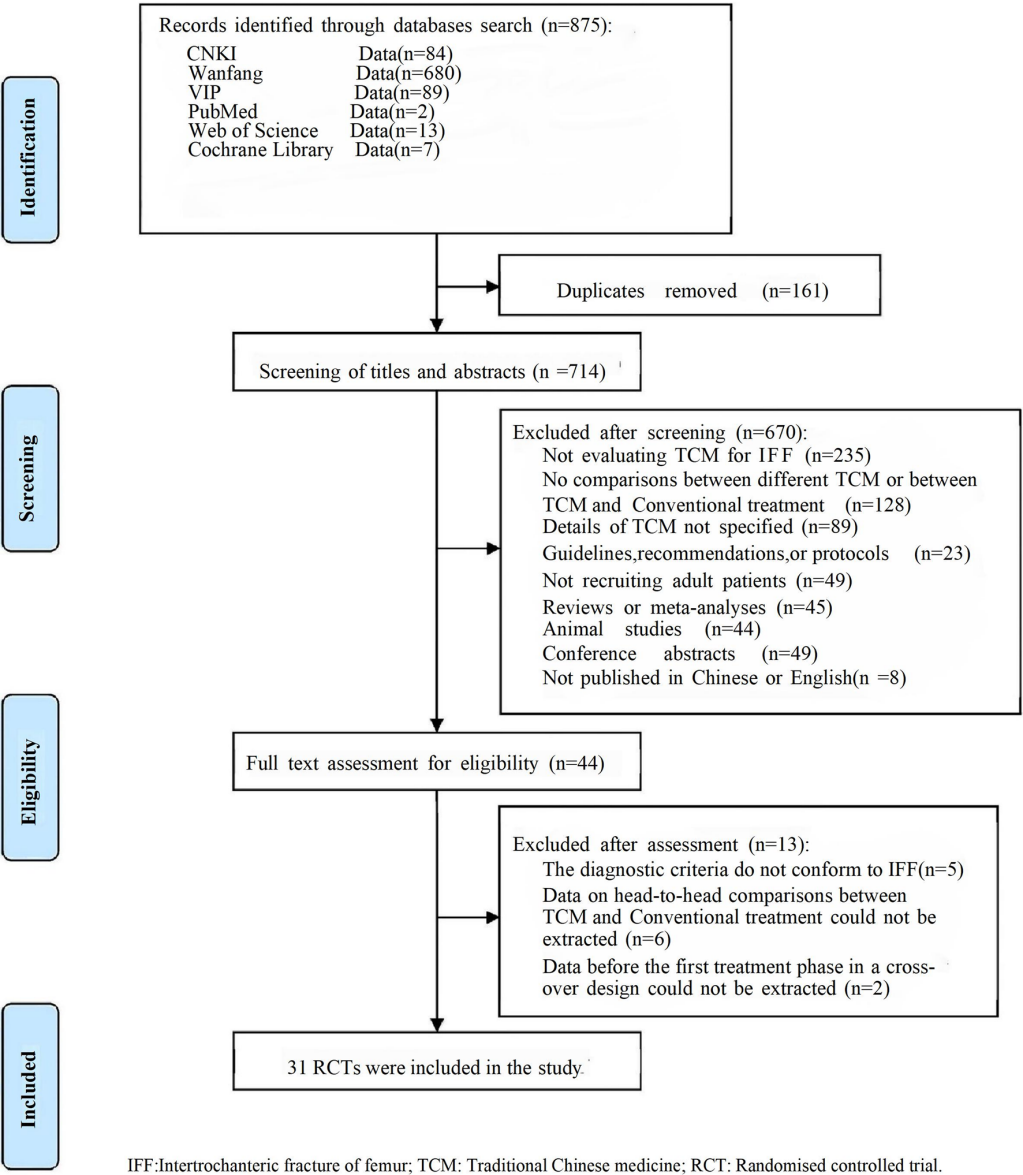
Flow chart of the search for eligible RCTs

A total of 875 pieces of literature were retrieved, with 31 ultimately included after screening, consisting of 1 four-arm and 30 two-arm trials [20–55]. Figure 2 illustrates the screening procedure. The studies involved 2076 individuals diagnosed with IFF, with 1041 in the experimental group and 1035 in the control group. Seventeen types of CHM were included, such as Bazhen decoction, Buxue Quyu decoction, Buyang Huanwu decoction, Danggui Buxue decoction, Fuyuan decoction, Guipi decoction, Shiquan Dabu decoction, Yangxue Rougan decoction, Qili Powder, Qitian Keli prescription, Sanqi powder, Shengxue Busui decoction, Shengyu decoction, Siwu decoction, Taohong Siwu decoction, and Yiqi Buxue decoction. The control group received conventional treatment, while the observation group received a combination of conventional treatment and CHM. Table 1 presents the fundamental characteristics of the included studies.

**Table 1:** Characteristics of the included studies

Study ID	AGE (YERS)		SEX (M/F)		N		Intervention		Cs/day	Outcome
	E	C	E	C	E	C	E	C		
Liu et al. [20]	73.6 ± 7.1	72.8 ± 6.5	20/14	19/15	34	34	CT + Bazhen decoction	CT	14	1, 3
Zhong [21]	72.13 ± 7.35	73.43 ± 6.04	13/17	12/18	30	30	CT + Bazhen decoction	CT	7	1, 2, 4
Zhu et al. [22]	70.75 ± 8.84	70.54 ± 8.96	22/19	20/21	41	41	CT + Bazhen decoction	CT	28	1, 2, 4
Hu et al. [25]	68.98 ± 7.12	67.34 ± 6.34	24/16	23/17	40	40	CT + Bazhen decoction	CT	14	1
Xie [37]	77.2 ± 10.11	78.8 ± 8.35	7/13	7/13	20	20	CT + Buxue Quyu decoction	CT	5	3
Xia et al. [23]	58.68 ± 5.43	59.24 ± 5.52	24/18	22/20	42	42	CT + Buyang Huanwu decoction	CT	14	2
Huang et al. [28]	77.9 ± 7.2	79.2 ± 8.6	20/14	18/16	34	34	CT + Danggui Buxue decoction	CT	7	1, 3
Yu (2022)	66.63 ± 5.79	64.67 ± 6.07	10/20	8/22	30	30	CT + Danggui Buxue decoction	CT	7	1, 4
Deng et al. [38]	72 ± 8	72 ± 7	14/16	13/17	30	30	CT + Danggui Buxue decoction	CT	7	1, 3, 4
Xing [39]	79.67 ± 7.74	78.33 ± 8.93	14/16	12/18	30	30	CT + Fuyuan decoction	CT	7	1, 4
Zhang et al. [19]	71.8 ± 2.8	70.9 ± 5.5	20/20	22/18	40	40	CT + Danggui Buxue decoction	CT	3	4
Wei [26]	80 ± 7.51	79 ± 6.94	3/12	4/11	15	15	CT + Guipi decoction	CT	7	1
Xiong et al. [24]	78.35 ± 5.08	79.05 ± 5.03	12/22	13/21	34	34	CT + Bazhen decoction	CT	7	2
Shi [40]	76.03 ± 0.98	75.43 ± 1.06	13/16	13/15	29	28	CT + Bazhen decoction	CT	7	1, 2
Jiang et al. [41]	61–80	62–78	34/26	33/27	60	60	CT + Buyang Huanwu decoction	CT	14	4
Hao et al. [42]	81.13 ± 7.53	83.00 ± 6.47	18/12	14/16	30	30	CT + Danggui Buxue decoction	CT	14	3
Li et al. [43]	71.47 ± 4.66	72.06 ± 3.49	38/22	35/25	60	60	CT + Shiquan Dabu decoction	CT	10	1, 2
Xu [44]	73.08 ± 7.08	72.80 ± 6.98	12/28	14/26	39	35	CT + Yangxue Rougan decoction	CT	7	1, 2, 4
Guo [27]	80 ± 6.33	80。67 ± 7.65	5/10	6/9	15	15	CT + Qili powder	CT	14	1
Cai [45]	81.23 ± 3.169	80.30 ± 3.271	11/19	14/16	30	30	CT + Qitian Keli prescription	CT	7	1, 2
Li [46]	75.73 ± 9.62	76.51 ± 9.53	NW	NW	30	30	CT + Sanqi powder	CT	7	1, 2
Song et al. [30]	67.28 ± 3.65	66.75 ± 3.82	18/22	16/24	40	40	CT + Sanqi powder	CT	7	1, 2, 3, 4
Zhou [47]	80.50 ± 8.60	77.86 ± 9.45	14/16	13/17	30	30	CT + Shengxue Busui decoction	CT	2	4
Yao [48]	77.57 ± 1.61	77.66 ± 1.63	8/22	9/20	30	29	CT + Shengyu decoction	CT	12	4
Ai et al. [49]	83.6 ± 7.2	6.4 ± 4.2	18/22	16/24	40	40	CT + Shiquan Dabu decoction	CT	14	4
Jin et al. [50]	69.62 ± 10.9	76.42 ± 7.25	18/16	15/19	34	34	CT + Siwu decoction	CT	12	1, 2
Zhong et al. [51]	NW	NW	NW	NW	30	30	CT + Siwu decoction	CT	10	1, 2
Li [52]	71.37 ± 6.16	70.11 ± 5.43	15/12	12/16	30	30	CT + Taohong Siwu decoction	CT	7	1, 2, 4
Xu [53]	78.16 ± 6.53	77.83 ± 5.84	7/13	8/12	20	20	CT + Yiqi Buxue decoction	CT	14	1, 2
Fu et al. [54]	69.22 ± 8.03	69.98 ± 7.99	18/16	17/17	34	34	CT + Zhixue Buxue decoction	CT	7	4
Yang et al. [55]	69.36 ± 3.02	68.32 ± 2.34	20/20	24/16	40	40	CT + Guipi decoction	CT	14	1, 2, 4

1:HB; 2: hidden blood loss; 3:Harris score; 4: Incidence of adverse reactions; *C* control group; *E* experimental group; *F* female; *M* male; *N* number; *CT* conventional treatment; *Cs* Course

### Quality evaluation

**Fig. 3:**
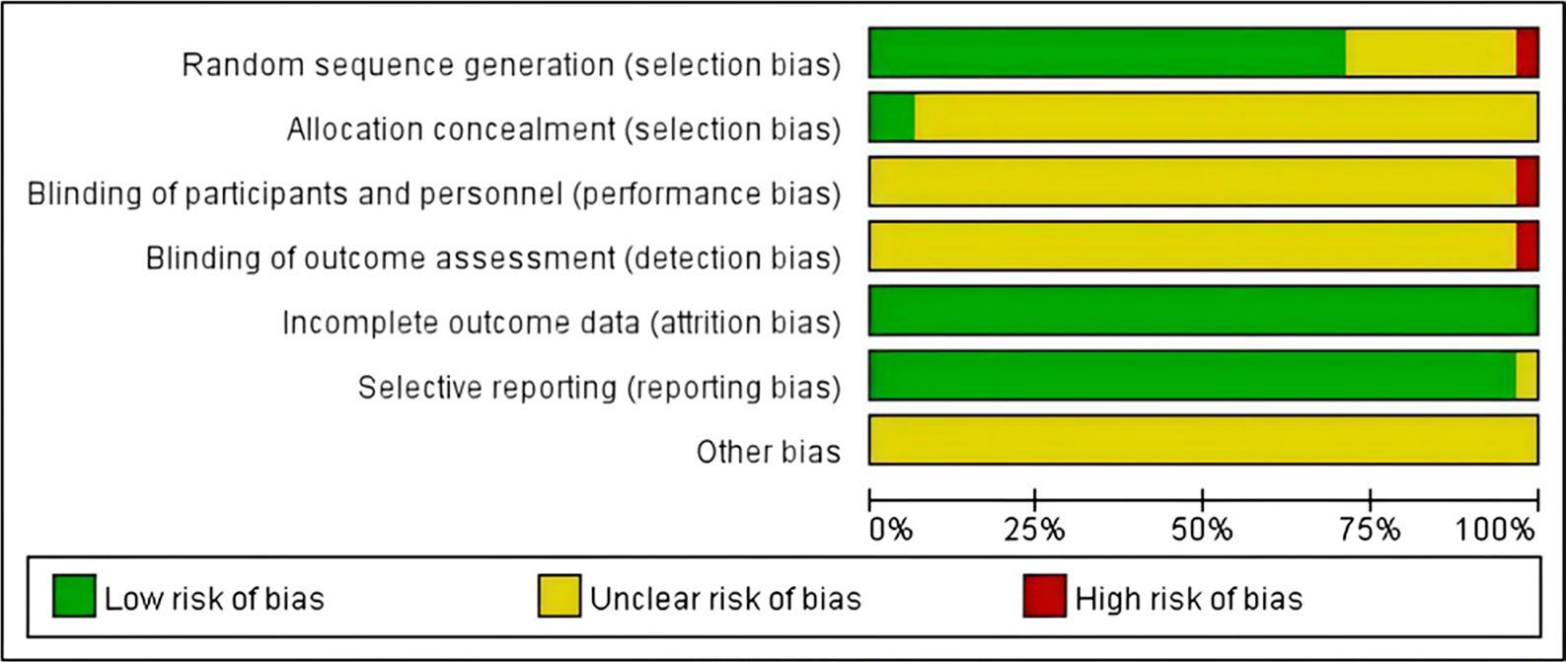
Risk-of-bias graph

All included studies were in Chinese. Regarding random sequence generation, 20 studies used the random number table technique, 1 used the coin toss method, and 1 used the two-colour ball method, all classified as low risk. One study was allocated randomly based on treatment sequence and classified as high risk; the remaining eight studies only mentioned randomness without detailing the method. Only 2 studies reported allocation concealment, while none mentioned blinding. All studies reported outcome measures except for one that omitted outcome indicators, resulting in a low dropout rate classified as low risk. No additional biases were reported. The findings are displayed in Fig. 3. The GRADE evaluation results showed that the Postoperative hidden blood loss and the incidence of adverse reactions were moderate quality evidence, and the Postoperative HB and Postoperative Harris score were low quality evidence, as shown in Table 2.

**Table 2:** GRADE evidence quality grading of each outcome indicator

Outcome indicator	Sample size		Risk of bias	Inconsistency	Indirectness	Inaccuracy	Publication bias	Quality of evidence	Grade
	E	C							
Postoperative HB	681	676	Serious^a^	Very serious^b^	Serious^c^	None	None	□□○○	**Low**
Postoperative hidden blood loss	529	524	Serious^a^	Serious^b^	Serious^c^	None	Existence	□□□○	**Medium**
Postoperative Harris score	188	188	Serious^a^	Serious^b^	Serious^c^	None	None	□□○○	**Low**
Incidence of adverse reactions	544	539	Serious^a^	None	Serious^c^	None	Existence	□□□○	**Medium**

Meaningful data were tabulated in bold

*C* control group; *E* experimental group

^a^Blinding is missing and allocation concealment is inadequate

^b^High heterogeneity

^c^Lack of head-to-head comparisons

## Outcomes

### Postoperative HB

#### Evidence network

**Fig. 4:**
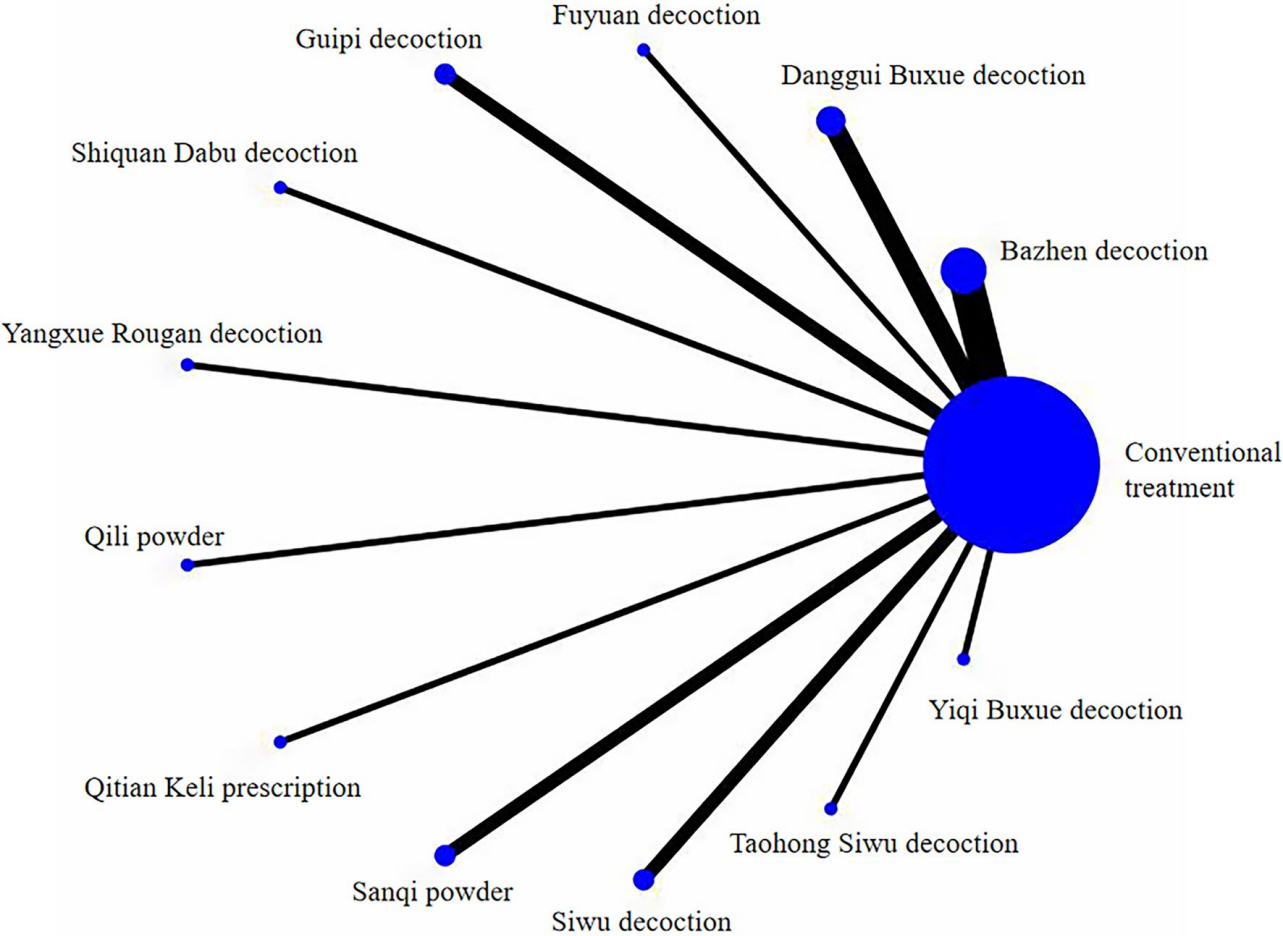
Evidence network of postoperative HB

Twenty-one studies reported postoperative HB values, including 20 two-arm studies and 1 four-arm study, encompassing 12 interventions. The overall evidence network was centred on conventional treatment to form a cross-comparison, as illustrated in Fig. 4.

### Traditional meta-analysis

Upon amalgamating the data, substantial heterogeneity was evident (*P* < 0.00001, I^2^ = 91%). To address this, subgroup analyses based on CHM categories were conducted, resulting in a significant reduction in heterogeneity. The traditional meta-analysis revealed that the combination of conventional treatment with Danggui Buxue decoction, Sanqi powder, Siwu decoction, Guipi decoction, Shiquan Dabu decoction, Qitian Keli prescription, Taohong Siwu decoction, Yiqi Buxue decoction, Fuyuan decoction, and Yangxue Rougan decoction had a more favourable effect on postoperative HB compared to conventional treatment alone (Table 3).

**Table 3:** Traditional meta-analysis of postoperative HB

Intervention	N	MD (95%CI)	*P*	Heterogeneity test
CT + Bazhen decoction vs CT	5	3.34 (− 3.31, 9.98)	0.33	***P* < 0.00001 I**^**2**^** = 97%**
CT + Danggui Buxue decoction vs CT	3	7.47 (3.38, 11.55)	**0.0003**	***P* = 0.03 I**^**2**^** = 72%**
CT + Sanqi powder vs CT	2	11.87 (6.32, 17.41)	** < 0.0001**	***P* = 0.11 I**^**2**^** = 62%**
CT + Siwu decoction vs CT	2	9.12 (7.41, 10.83)	** < 0.00001**	***P* = 0.79 I**^**2**^** = 0%**
CT + Guipi decoction vs CT	2	9.89 (4.07, 15.71)	**0.0009**	***P* = 0.76 I**^**2**^** = 0%**
CT + Shiquan Dabu decoction vs CT	1	10.44 (6.03, 14.85)	** < 0.00001**	
CT + Qili powder vs CT	1	2.23 (− 1.88, 6.34)	0.29	
CT + Qitian Keli prescription vs CT	1	5.33 (2.66, 8.00)	** < 0.0001**	
CT + Taohong Siwu decoction vs CT	1	6.56 (0.59, 12.53)	**0.03**	
CT + Yiqi Buxue decoction vs CT	1	14.92 (7.33, 22.51)	**0.0001**	
CT + Fuyuan decoction vs CT	1	5.70 (2.59, 8.81)	**0.0003**	
CT + Yangxue Rougan decoction vs CT	1	13.50 (8.07, 18, 93)	** < 0.00001**	
Total	21	7.37 (4.89, 9.84)	** < 0.00001**	***P* < 0.00001 I**^**2**^** = 91%**

Meaningful data were tabulated in bold

*N* number; *CT* conventional treatment

### Network meta-analysis

Ninety-one reciprocal comparisons were established in total. The network meta-analysis results indicated that, in terms of postoperative HB, conventional treatment combined with Yiqi Buxue decoction, Yangxue Rougan decoction, Sanqi powder, Guipi decoction, Siwu decoction, and Danggui Buxue decoction was significantly more effective than conventional treatment alone (*P* < 0.05). The effectiveness of other proprietary CHM showed no significant difference (*P* > 0.05) (Table 4).

**Table 4:** Network meta-analysis of postoperative HB

Intervention	Yiqi Buxue decoction	Yangxue Rougan decoction	Sanqi powder	Shiquan Dabu decoction	Guipi decoction	Siwu decoction	Danggui Buxue decoction	Taohong Siwu decoction	Fuyuan decoction	Qitian Keli prescription	Bazhen decoction	Qili powder	conventional treatment
Yiqi Buxue decoction	0												
Yangxue Rougan decoction	1.42 (− 15.62, 18.46)	0											
Sanqi powder	2.78 (− 12.12, 17.67)	1.36 (− 12.56,15.28)	0										
Shiquan Dabu decoction	4.48 (− 12.27, 21.23)	3.06 (− 12.83, 18.95)	1.70 (− 11.85, 15.26)	0									
Guipi decoction	4.89 (− 10.78, 20.56)	3.47 (− 11.27, 18.21)	2.11 (− 10.08, 14.31)	0.41 (− 13.99, 14.81)	0								
Siwu decoction	5.57 (− 9.15, 20.28)	4.15 (− 9.58, 17.87)	2.79 (− 8.16, 13.73)	1.09 (− 12.27, 14.44)	0.68 (− 11.30, 12.65)	0							
Danggui Buxue decoction	7.46 (− 6.61, 21.53)	6.04 (− 7.00, 19.07)	4.68 (− 5.39, 14.75)	2.98 (− 9.67, 15.62)	2.57 (− 8.61, 13.74)	1.89 (− 7.90, 11.69)	0						
Taohong Siwu decoction	8.36 (− 8.87, 25.59)	6.94 (− 9.45, 23.33)	5.58 (− 8.56, 19.72)	3.88 (− 12.20, 19.96)	3.47 (− 11.48, 18.42)	2.79 (− 11.16, 16.74)	0.90 (− 12.37, 14.17)	0					
Fuyuan decoction	9.22 (− 7.23, 25.67)	7.80 (− 7.77, 23.37)	6.44 (− 6.75, 19.63)	4.74 (− 10.51, 19.99)	4.33 (− 9.73, 18.38)	3.65 (− 9.33, 16.64)	1.76 (− 10.49, 14.01)	0.86 (− 14.91, 16.63)	0				
Qitian Keli prescription	9.59 (− 6.79, 25.97)	8.17 (− 7.32, 23.66)	6.81 (− 6.28, 19.90)	5.11 (− 10.06, 20.28)	4.70 (− 9.27, 18.66)	4.02 (− 8.86, 16.91)	2.13 (− 10.02, 14.28)	1.23 (− 14.46, 16.92)	0.37 (− 14.47, 15.21)	0			
Bazhen decoction	11.65 (− 1.83, 25.13)	10.23 (− 2.16, 22.62)	8.87 (− 0.34, 18.09)	7.17 (− 4.81, 19.15)	6.76 (− 3.65, 17.18)	6.09 (− 2.83, 15.01)	4.19 (− 3.62, 12.01)	3.29 (− 9.35, 15.93)	2.43 (− 9.13, 14.00)	2.06 (− 9.39, 13.52)	0		
Qili powder	12.69 (− 3.98, 29.36)	11.27 (− 4.54, 27.08)	9.91 (− 3.55, 23.37)	8.21 (− 7.28, 23.70)	7.80 (− 6.51, 22.11)	7.12 (− 6.14, 20.38)	5.23 (− 7.31, 17.78)	4.33 (− 11.67, 20.33)	3.47 (− 11.70, 18.64)	3.10 (− 11.98, 18.18)	1.04 (− 10.84, 12.91)	0	
conventional treatment	**14.92 (2.30, 27.54)**	**13.50 (2.05, 24.95)**	**12.14 (4.23, 20.05)**	10.44 (− 0.57, 21.45)	**10.03 (0.75, 19.31)**	**9.35 (1.79, 16.92)**	**7.46 (1.24, 13.69)**	6.56 (− 5.16, 18.28)	5.70 (− 4.85, 16.25)	5.33 (− 5.10, 15.76)	3.27 (− 1.46, 7.99)	2.23 (− 8.66, 13.12)	0

### Postoperative hidden blood loss

#### Evidence network

**Fig. 5:**
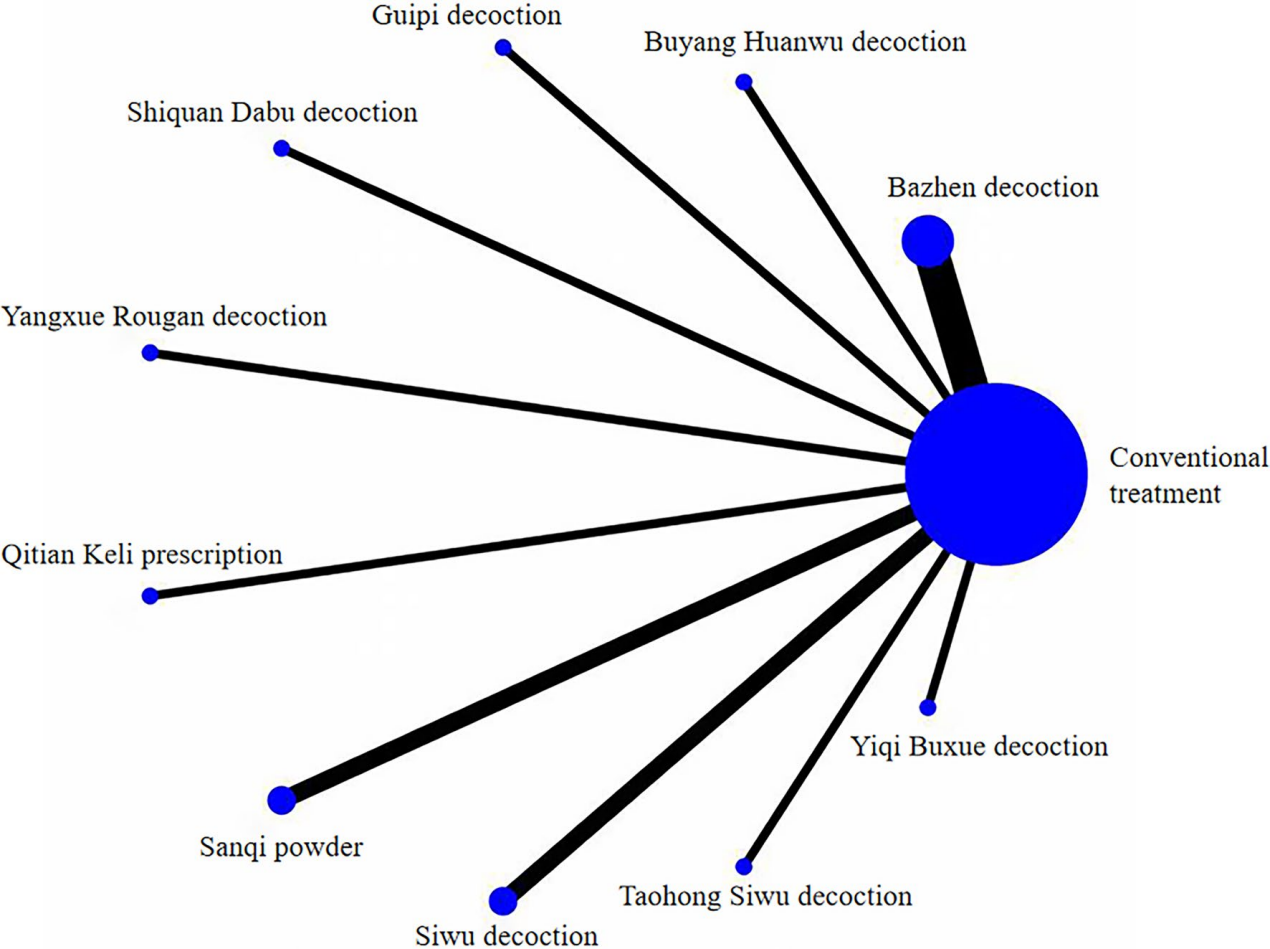
Evidence network of postoperative hidden blood loss

Fifteen studies reported the amount of hidden bleeding post-surgery, including 14 two-arm studies and 1 four-arm study, involving 10 interventions. The evidence network primarily centred on conventional care to facilitate cross-comparison, as depicted in Fig. 5.

### Traditional meta-analysis

Upon data amalgamation, substantial heterogeneity was observed (*P* = 0.0002, I^2^ = 66%). Segmentation into subgroups based on CHM types significantly reduced this heterogeneity. Traditional meta-analysis results indicated that combining conventional treatment with Bazhen decoction, Siwu decoction, Buyang Huanwu decoction, Guipi decoction, Shiquan Dabu decoction, Yangxue Rougan decoction, Qitian Granule decoction, Taohong Siwu decoction, and Yiqi Buxue decoction was more effective in reducing hidden bleeding post-surgery than conventional treatment alone (Table 5).

**Table 5:** Traditional meta-analysis of postoperative hidden blood loss

Intervention	N	MD (95%CI)	*P*	Heterogeneity test
CT + Bazhen decoction vs CT	4	− 52.46 (− 58.57, − 46.35)	** < 0.00001**	***P* = 0.30 I**^**2**^** = 18%**
CT + Sanqi powder vs CT	2	− 74.39 (− 184.35, − 35.57)	**0.18**	***P* = 0.03 I**^**2**^** = 78%**
CT + Siwu decoction vs CT	2	− 86.97 (− 103.04, − 70.90)	** < 0.00001**	***P* = 0.85 I**^**2**^** = 0%**
CT + Buyang Huanwu decoction vs CT	1	− 64.73 (− 81.53,− 47.93)	** < 0.00001**	
CT + Guipi decoction vs CT	1	− 47.00 (− 53.62, − 40.38)	** < 0.00001**	
CT + Shiquan Dabu decoction vs CT	1	− 57.68 (− 83.73, − 31.63)	** < 0.0001**	
CT + Yangxue Rougan decoction vs CT	1	− 69.90 (− 104.06, − 35.74)	** < 0.0001**	
CT + Qitian Keli prescription vs CT	1	− 74.27 (− 131.92, − 16.62)	**0.01**	
CT + Taohong Siwu decoction vs CT	1	− 42.44 (− 83.26, − 1.62)	**0.04**	
CT + Yiqi Buxue decoction vs CT	1	− 34.78 (− 59.06, − 10.50)	**0.005**	
Total	15	− 54.55 (− 63.75, − 45.35)	** < 0.00001**	***P* = 0.0002 I**^**2**^** = 66%**

Meaningful data were tabulated in bold

*N* number; *CT* conventional treatment

### Network meta-analysis

Sixty-six reciprocal comparisons were established. Network meta-analysis results demonstrated that, concerning postoperative hidden bleeding, the combination of conventional treatment with Siwu decoction, Yangxue Rougan decoction, Qitian Keli prescription, Buyang Huanwu decoction, Shiquan Dabu decoction, Bazhen decoction, Guipi decoction, Taohong Siwu decoction, Yiqi Buxue decoction, and Sanqi powder was superior to conventional treatment alone, with statistically significant differences (*P* < 0.05). Specifically, the combination with Siwu decoction was more effective than with Bazhen decoction, Guipi decoction, Taohong Siwu decoction, Yiqi Buxue decoction, and Sanqi powder (*P* < 0.05). The efficacy of combining Yangxue Rougan decoction with conventional treatment surpassed that of Sanqi powder (*P* < 0.05). Additionally, Buyang Huanwu decoction was more effective than Yiqi Buxue decoction and Sanqi powder (*P* < 0.05). The healing impact of Bazhen decoction exceeded that of Sanqi powder (*P* < 0.05). No notable variance was observed in the effectiveness of other proprietary CHMs (*P* > 0.05) (Table 6).

**Table 6:** Network meta-analysis of postoperative hidden blood loss

Intervention	Siwu decoction	Yangxue Rougan decoction	Qitian Keli prescription	Buyang Huanwu decoction	Shiquan Dabu decoction	Bazhen decoction	Guipi decoction	Taohong Siwu decoction	Yiqi Buxue decoction	Sanqi powder	Conventional treatment
Siwu decoction	0										
Yangxue Rougan decoction	− 17.07 (− 54.82, 20.68)	0									
Qitian Keli prescription	− 12.70 (− 72.55, 47.15)	4.37 (− 62.64, 71.38)	0								
Buyang Huanwu decoction	− 22.24 (− 45.49, 1.00)	− 5.17 (− 43.24, 32.90)	− 9.54 (− 69.59, 50.51)	0							
Shiquan Dabu decoction	− 29.29 (− 59.90, 1.31)	− 12.22 (− 55.18, 30.74)	− 16.59 (− 79.85, 46.67)	− 7.05 (− 38.04, 23.94)	0						
Bazhen decoction	− **34.51 (**− **51.70, **− **17.32)**	− 17.44 (− 52.15, 17.26)	− 21.81 (− 79.79, 36.16)	− 12.27 (− 30.15, 5.60)	− 5.22 (− 31.98, 21.53)	0					
Guipi decoction	− **39.97 (**− **57.35, **− **22.60)**	− 22.90 (− 57.69, 11.89)	− 27.27 (− 85.30, 30.76)	− 17.73 (− 35.79, 0.33)	− 10.68 (− 37.55, 16.19)	− 5.46 (− 14.46, 3.55)	0				
Taohong Siwu decoction	− **44.53 (**− **88.40, **− **0.67)**	− 27.46 (− 80.69, 25.77)	− 31.83 (− 102.47, 38.81)	− 22.29 (− 66.43, 21.85)	− 15.24 (− 63.66, 33.18)	− 10.02 (− 51.29, 31.25)	− 4.56 (− 45.91, 36.79)	0			
Yiqi Buxue decoction	− **52.19 (**− **81.31, **− **23.07)**	− 35.12 (− 77.03, 6.79)	− 39.49 (− 102.05, 23.07)	− **29.95 (**− **59.48, **− **0.42)**	− 22.90 (− 58.51, 12.71)	− 17.68 (− 42.72, 7.36)	− 12.22 (− 37.39, 12.95)	− 7.66 (− 55.15, 39.83)	0		
Sanqi powder	− **55.44 (**− **77.87, **− **33.01)**	− **38.37 (**− **75.94, **− **0.80)**	− 42.74 (− 102.47, 17.00)	− **33.20 (**− **56.15, **− **10.24)**	− 26.15 (− 56.53, 4.24)	− **20.92 (**− **37.72, **− **4.13)**	− 15.47 (− 32.45, 1.52)	− 10.91 (− 54.62, 32.80)	− 3.25 (− 32.13, 25.64)	0	
conventional treatment	− **86.97 (**− **103.04, **− **70.90)**	− **69.90 (**− **104.06, **− **35.74)**	− **74.27 (**− **131.92, **− **16.62)**	− **64.73 (**− **81.53, **− **47.93)**	− **57.68 (**− **83.73, **− **31.63)**	− **52.46 (**− **58.57, **− **46.35)**	− **47.00 (**− **53.62, **− **40.38)**	− **42.44 (**− **83.26, **− **1.62)**	− **34.78 (**− **59.06, **− **10.50)**	− **31.53 (**− **47.18, **− **15.89)**	0

Meaningful data were tabulated in bold

### Postoperative Harris score

#### Evidence network

**Fig. 6:**
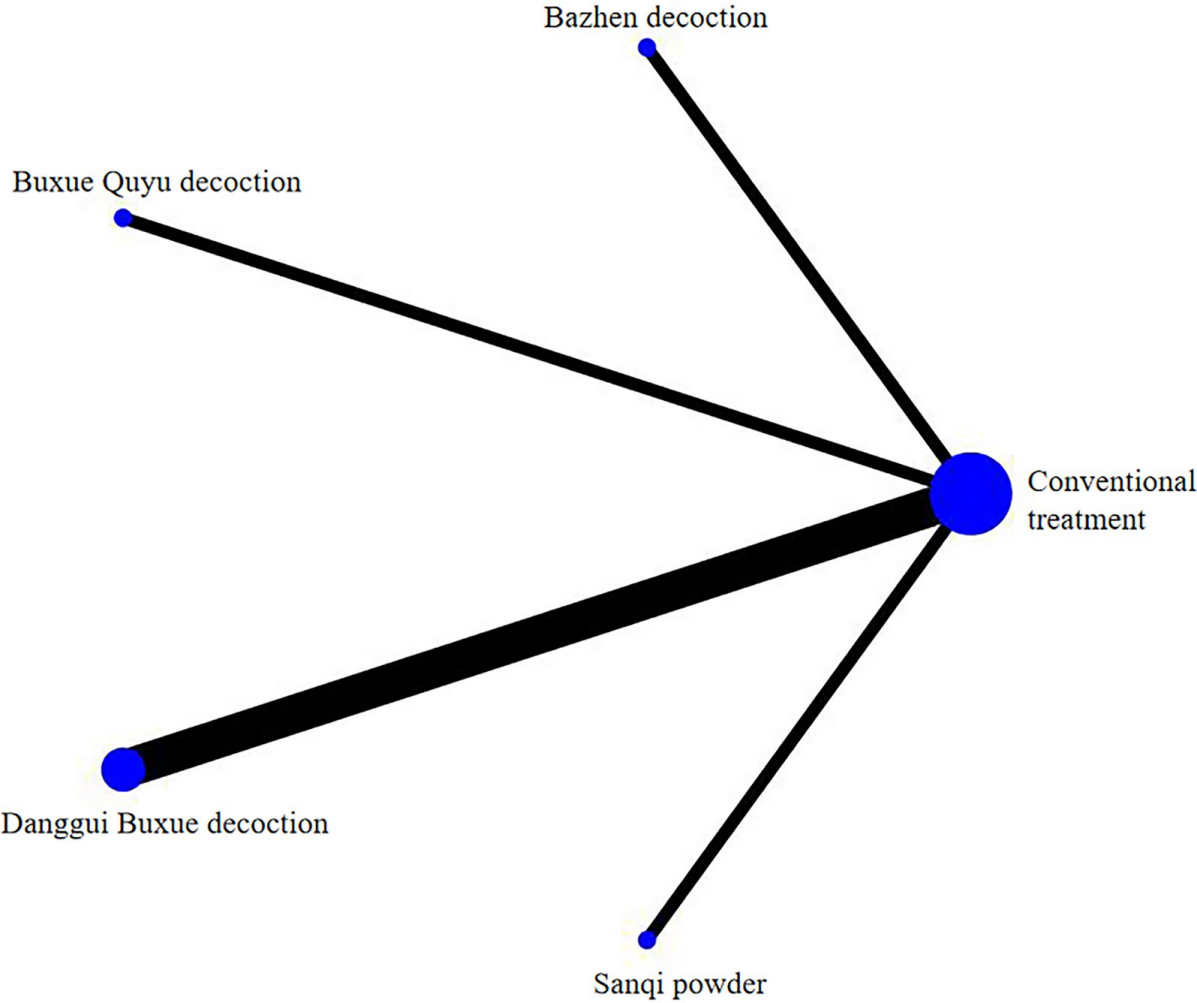
Evidence network of postoperative Harris score

Six studies reported postoperative Harris scores, all of which were two-arm studies involving four interventions. The overall evidence network was centred on conventional treatment for comparison, as shown in Fig. 6.

### Traditional meta-analysis

Significant heterogeneity across the studies (*P* < 0.00001, I^2^ = 87%) was observed after data amalgamation. Subgrouping the data according to CHM types significantly reduced this heterogeneity. Traditional meta-analysis results indicated that conventional treatment combined with Danggui Buxue decoction and Bazhen decoction significantly improved hip joint function post-surgery compared to conventional treatment alone (Table 7).

**Table 7:** Traditional meta-analysis of postoperative Harris score

Intervention	N	MD (95%CI)	*P*	Heterogeneity test
CT + Danggui Buxue decoction vs CT	3	3.61 (2.22, 5.01)	** < 0.00001**	***P* = 0.61 I**^**2**^** = 0%**
CT + Bazhen decoction vs CT	1	10.62 (4.88, 16.36)	**0.0003**	
CT + Buxue Quyu decoction vs CT	1	− 0.40 (− 1,02, 0.22)	0.21	
CT + Sanqi powder vs CT	1	0.21 (− 1.32, 1.74)	0.79	
Total	6	2.80 (0.61, 4.98)	**0.01**	***P* < 0.00001 I**^**2**^** = 87%**

Meaningful data were tabulated in bold

*N* number; *CT* conventional treatment

### Network meta-analysis

Fifteen comparative analyses conducted via network meta-analysis revealed that, in terms of postoperative Harris scores, conventional treatment combined with Bazhen decoction outperformed combinations with Danggui Buxue decoction, Sanqi powder, Buxue Quyu decoction, and conventional treatment alone (*P* < 0.05). Similarly, the combination of conventional treatment with Danggui Buxue decoction was more effective than combinations with Sanqi powder, Buxue Quyu decoction, and conventional treatment alone (*P* < 0.05). Other proprietary CHMs showed no significant difference in effectiveness (*P* > 0.05) (Table 8).

**Table 8:** Network meta-analysis of postoperative Harris score

Intervention	Bazhen decoction	Danggui Buxue decoction	Sanqi powder	Conventional treatment	Buxue Quyu decoction
Bazhen decoction	0				
Danggui Buxue decoction	**7.01 (1.10, 12.92)**	0			
Sanqi powder	**10.41 (4.47, 16.35)**	**3.40 (1.33, 5.48)**	0		
conventional treatment	10.62 (4.88, 16.36)	3.61 (2.22, 5.01)	0.21 (− 1.32, 1.74)	0	
Buxue Quyu decoction	**11.02 (5.24, 16.80)**	**4.01 (2.49, 5.54)**	0.61 (− 1.04, 2.26)	0.40 (− 0.22, 1.02)	0

Meaningful data were tabulated in bold

### Incidence of adverse reactions

Out of the 31 reviewed studies, 15 reported negative effects. Seven studies noted no adverse reactions in either the experimental or control groups, while four reported specific adverse effects such as nausea, diarrhoea, rash, headache, dizziness, irregular liver function, and coagulation issues. Adverse reactions in five cases were attributed to surgical and non-CHM factors, including incision complications, deep venous thrombosis of the lower extremities, pulmonary infection, hemorrhagic anaemia, hypoproteinemia, and other systemic complications. No adverse effects were reported in the remaining 16 studies (Table 9).

**Table 9:** Occurrence of adverse reactions

Group	Reports of adverse reactions	N	Ae/c(T/C)	Aet/%(T/C)
CT + Shiquan Dabu decoction	**Pulmonary infection 7 c, deep venous thrombosis of lower extremity 4 c**	**40**	**11/18**	**27.5/45**
CT	**Pulmonary infection 12 c, deep venous thrombosis of lower extremity 6 c**	**40**		
CT + Yangxue Rougan decoction	**Diarrhea 3 c**	**40**	**3/1**	**7.5/2.5**
CT	**Headache and dizziness 1 c**	**40**		
CT + Danggui Buxue decoction	**Incision complication 1 c, systemic complications 1 c**	**30**	**2/7**	**6.7/23.3**
CT	**Incision complication 1 c, deep venous thrombosis of lower extremity 4 c, systemic complications 2 c**	**30**		
CT + Taohong Siwu decoction	**Diarrhea 1 c, abnormal liver function 1 c**	**30**	**2/0**	**6.7/0**
CT	No	**30**		
CT + Bazhen decoction	**Nausea 1 c, rash 1 c**	**41**	**2/4**	**4.88/9.76**
CT	**Nausea 2 c, diarrhea 1 c、rash 1 c**	**41**		
CT + Buyang Huanwu decoction	**Deep venous thrombosis of lower extremity 2 c**	**60**	**2/7**	**3.33/11.67**
CT	**Deep venous thrombosis of lower extremity 7 c**	**60**		
CT + Zhixue Buxue decoction	**Hemorrhagic anemia 1 c**	**34**	**1/6**	**2.9/17.6**
CT	**Hemorrhagic anemia 2 c, hypoproteinemia 1 c, coagulation disorders 2 c, deep venous thrombosis of lower extremity 1 c**	**34**		
CT + Sanqi powder	No	**40**	**0/1**	**0/2.5**
CT	**Deep venous thrombosis of lower extremity 1 c**	**40**		
CT + Bazhen decoction	No	30	0/0	0/0
CT	No	30		
CT + Danggui Buxue decoction	No	30	0/0	0/0
CT	No	30		
CT + Danggui Buxue decoction	No	34	0/0	0/0
CT	No	34		
CT + Fuyuan decoction	No	30	0/0	0/0
CT	No	30		
CT + Shengxue Busui decoction	No	30	0/0	0/0
CT	No	30		
CT + Shengyu decoction	No	30	0/0	0/0
CT	No	29		
CT + Guipi decoction	No	15	0/0	0/0
CT	No	15		

Meaningful data were tabulated in bold

*N* number; *Ae* Adverse event; *Aet* Adverse event rate; *T* experimental group; *C* control group; *CT* conventional treatment; *c* case

### SUCRA values and sorting results

**Fig. 7:**
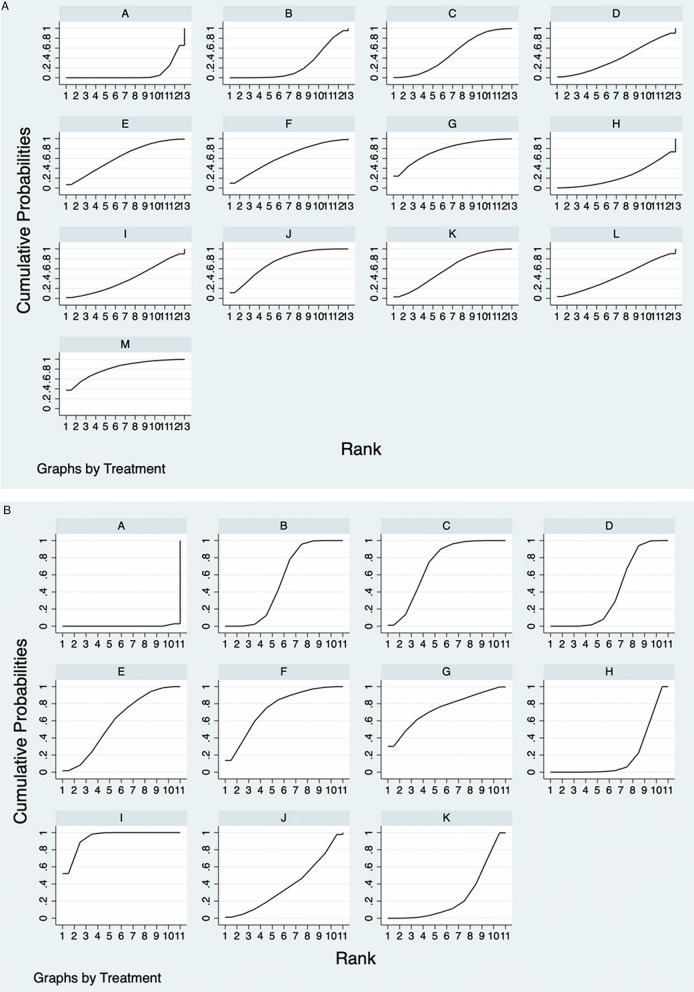

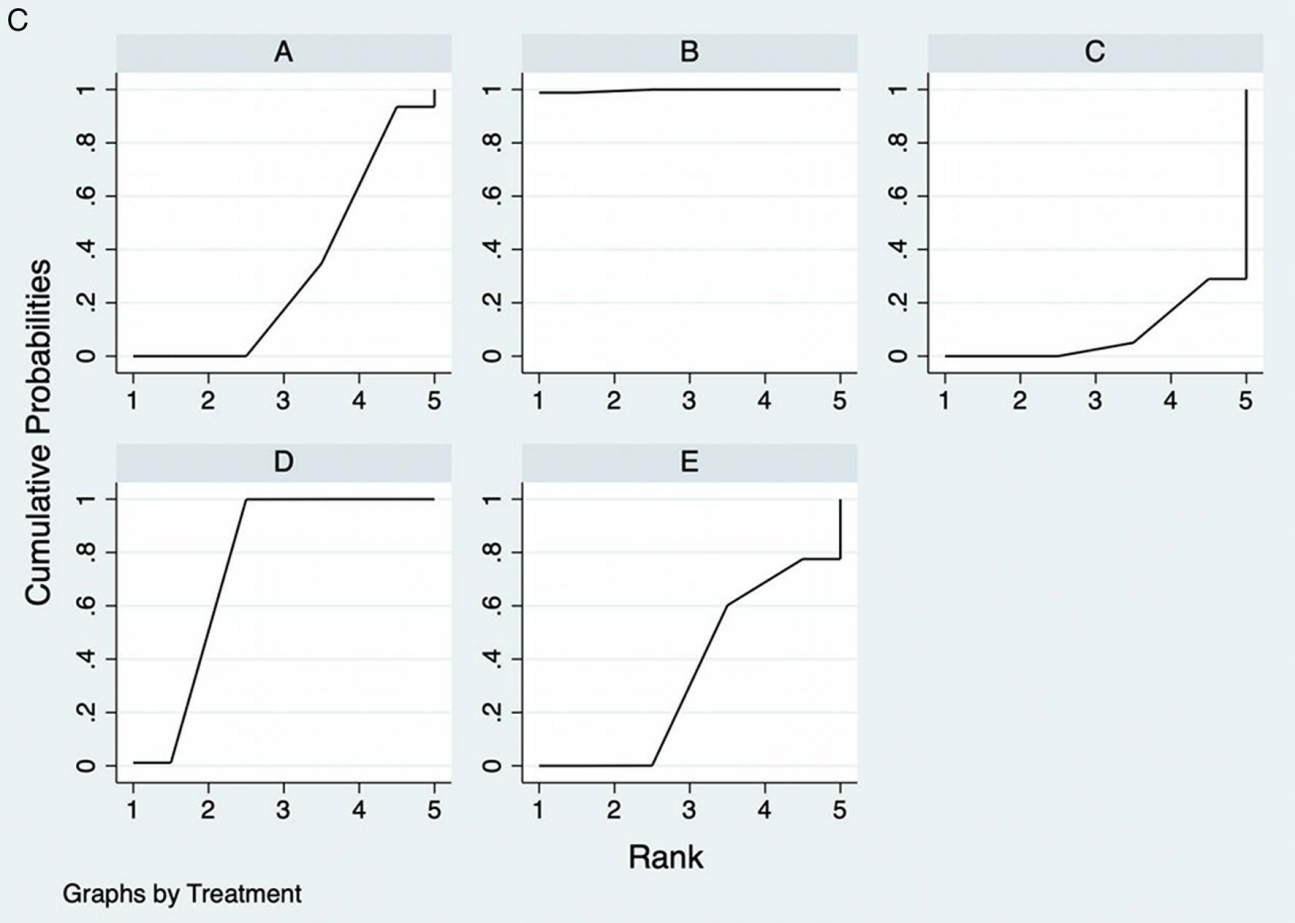
**A** Plot of the surface under the cumulative ranking curves for outcomes. Postoperative HB:A = conventional treatment, B = Bazhen decoction, C = Danggui Buxue decoction, D = Fuyuan decoction, E = Guipi decoction, F = Shiquan Dabu decoction, G = Yangxue Rougan decoction, H = Qili powder, I = Qitian Keli prescription, J = Sanqi powder, K = Siwu decoction, L = Taohong Siwu decoction, M = Yiqi Buxue decoction. **B** Plot of the surface under the cumulative ranking curves for outcomes. Postoperative hidden blood loss:A = conventional treatment, B = Bazhen decoction, C = Buyang Huanwu decoction, D = Guipi decoction, E = Shiquan Dabu decoction, F = Yangxue Rougan decoction, G = Qitian Keli prescription, H = Sanqi powder, I = Siwu decoction, J = Taohong Siwu decoction, K = Yiqi Buxue decoction. **C** Plot of the surface under the cumulative ranking curves for outcomes. Postoperative Harris score:A = conventional treatment, B = Bazhen decoction, C = Buxue Quyu decoction, D = Danggui Buxue decoction, E = Sanqi powder

Table 10, Fig. 7A–C displayed the SUCRA figures and the ranking outcomes for each outcome measure. The larger the SUCRA values, the more effective the intervention measures are. In terms of improving HB after operation, Yiqi Buxue decoction > Yangxue Rougan decoction > Sanqi Powder > Shiquan Da Bu decoction > Guipi decoction > Siwu decoction > Danggui Buxue decoction > Taohong Siwu decoction > Fuyuan decoction > Qitian Keli prescription > Bazhen decoction > Qili San > conventional treatment; In terms of reducing the amount of hidden bleeding after operation, Siwu decoction > Yangxue Rougan decoction > Qitian Keli prescription > Buyang Huanwu decoction > Shiquan Dabu decoction > Bazhen decoction > Guipi decoction > Taohong Siwu decoction > Yiqi Buxue decoction > Sanqi Powder > conventional treatment; In terms of improving postoperative Harris score, Bazhen decoction > Danggui Buxue decoction > Sanqi Powder > conventional treatment > Buxue Quyu decoction.

**Table 10:** SUCRA values and rank of network Meta-analysis

Intervention	HB		Hidden blood loss		Harris score	
	SUCRA	Sequence	SUCRA	Sequence	SUCRA	Sequence
Conventional treatment	8.0	**13**	0.3	**11**	32.1	**4**
Bazhen decoction	25.5	**11**	53.0	**6**	99.7	**1**
Danggui Buxue decoction	49.7	**7**			75.3	**2**
Fuyuan decoction	40.2	**9**				
Guipi decoction	62.9	**5**	40.0	**7**		
Shiquan Dabu decoction	63.2	**4**	59.5	**5**		
Yangxue Rougan decoction	76.8	**2**	74.9	**2**		
Qili powder	24.8	**12**				
Qitian Keli prescription	39.1	**10**	74.0	**3**		
Sanqi powder	74.1	**3**	19.3	**10**	34.5	**3**
Siwu decoction	59.8	**6**	93.9	**1**		
Taohong Siwu decoction	44.3	**8**	38.1	**8**		
Yiqi Buxue decoction	81.6	**1**	25.2	**9**		
Buyang Huanwu decoction			71.7	**4**		
Buxue Quyu decoction					8.5	**5**

Meaningful data were tabulated in bold

### Publication bias

**Fig. 8:**
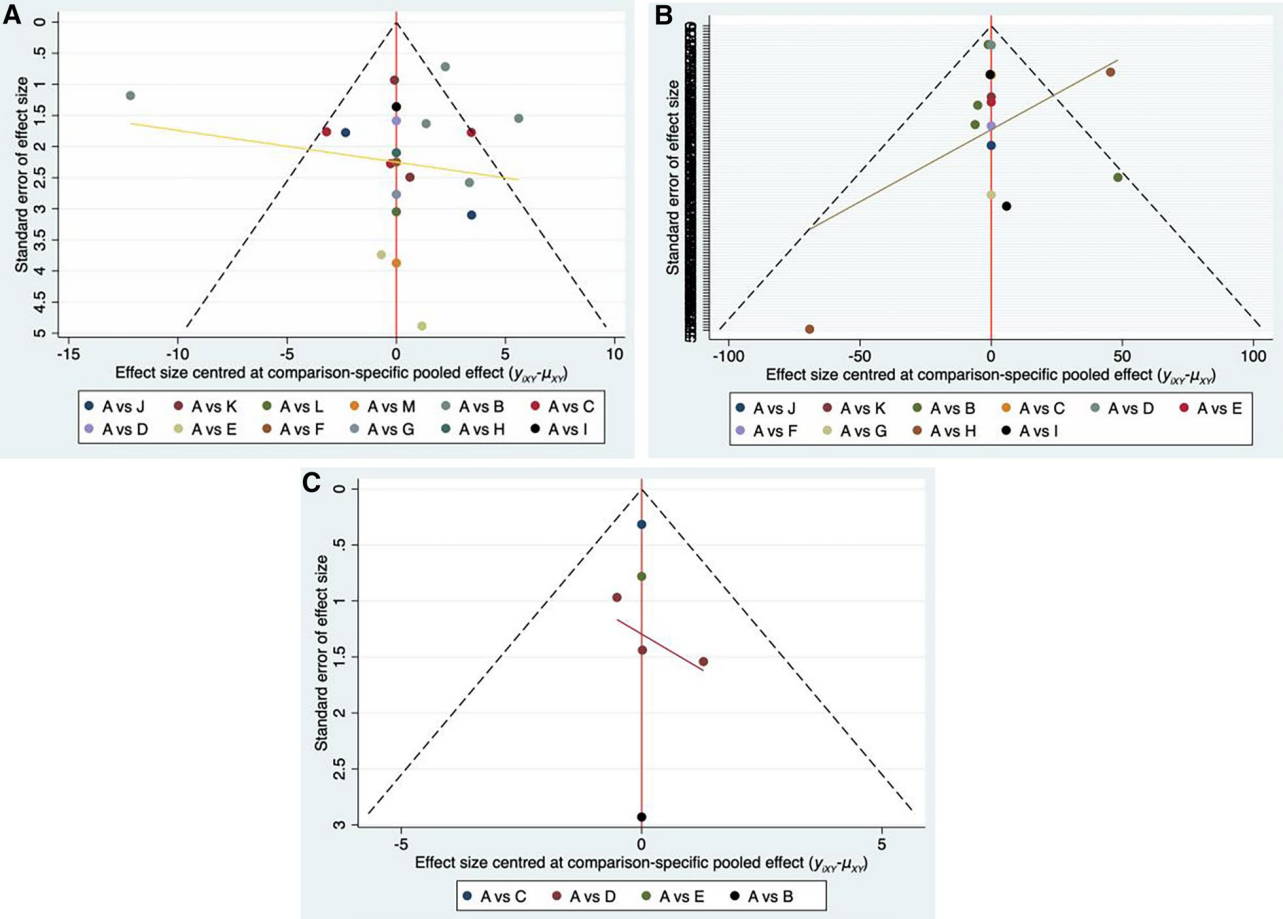
**A** Comparison-correction funnel plot of outcome indicators. Postoperative HB: A = conventional treatment, B = Bazhen decoction, C = Danggui Buxue decoction, D = Fuyuan decoction, E = Guipi decoction, F = Shiquan Dabu decoction, G = Yangxue Rougan decoction, H = Qili powder, I = Qitian Keli prescription, J = Sanqi powder, K = Siwu decoction, L = Taohong Siwu decoction, M = Yiqi Buxue decoction. **B** Comparison-correction funnel plot of outcome indicators. Postoperative hidden blood loss:A = conventional treatment, B = Bazhen decoction, C = Buyang Huanwu decoction, D = Guipi decoction, E = Shiquan Dabu decoction, F = Yangxue Rougan decoction, G = Qitian Keli prescription, H = Sanqi powder, I = Siwu decoction, J = Taohong Siwu decoction, K = Yiqi Buxue decoction. **C** Comparison-correction funnel plot of outcome indicators. Postoperative Harris score: A = conventional treatment, B = Bazhen decoction, C = Buxue Quyu decoction, D = Danggui Buxue decoction, E = Sanqi powder

Using Stata17.0, the impact of each outcome indicator on small samples was assessed, resulting in the creation of a "comparison-correction" funnel plot, depicted in Fig. 8A–C. The balanced funnel plot representations of HB, hidden blood loss, and Harris scores suggested a lower probability of publication bias.

## Discussion

The concept of hidden blood loss was first introduced by Sehat, who observed that haemoglobin levels in patients undergoing total knee replacement were consistently lower than the estimated intraoperative bleeding [7]. Analysis of 63 patients with total knee replacement revealed that total blood loss comprised both overt and hidden bleeding. Through a retrospective analysis of patients with intra-articular and extra-articular fractures, Smith et al. found that preoperative haemoglobin levels were significantly higher in intra-articular fractures compared to extra-articular fractures. Furthermore, the decline in haemoglobin from admission to surgery was greater for extra-articular fractures, indicating higher preoperative blood loss in these cases [56, 57]. Extra-articular fractures, including IFF and subtrochanteric fractures of the femur, are not restricted by a joint capsule, making them more likely to damage blood vessels and cause continuous bleeding along tissue spaces. IFF, in particular, involves the fracture line in the cancellous bone with a rich blood supply, leading to substantial bleeding and severe anaemia. Currently, prevention and treatment of postoperative hidden bleeding in IFF include blood transfusion, iron supplementation, antifibrinolytic drugs, and functional exercise [10]. Lawrence et al. demonstrated that postoperative blood transfusion in IFF significantly improves postoperative function and accelerates metabolism [58]. Autologous and allogeneic blood transfusions can rapidly improve HB levels, but they do not fundamentally address or prevent hidden blood loss [11]. Iron, as a hematopoietic raw material, promotes haemoglobin synthesis and red blood cell maturation, but it also merely enhances blood production without preventing hidden blood loss [12]. Early postoperative rehabilitation exercises improve lower limb circulation, enhance muscle strength, and promote blood infiltration and absorption within tissues, yet their effect on reducing hidden blood loss is minimal [13]. Antifibrinolytic drugs, such as tranexamic acid, primarily function through an anti-fibrinolytic enzyme. This enzyme strongly adsorbs to the lysine-binding sites on plasmin and plasminogen, inhibiting their binding to fibrin. This inhibition significantly prevents the decomposition of fibrin caused by plasmin. Moreover, in the presence of α2-macroglobulin and other anti-fibrinolytic enzymes in serum, the anti-fibrinolytic effect of these drugs becomes more pronounced, enhancing their hemostatic efficacy [14]. However, tranexamic acid has a risk of deep vein thrombosis [15], and the hemostatic effect of tranexamic acid alone is not significant. Many studies have shown that the combination of CHM and tranexamic acid has better efficacy. CHM, after complex processing and compatibility with various drugs, offers the advantages of minimal side effects and stable therapeutic outcomes. The mechanism of action of CHM is well-documented, with pharmacological studies highlighting the roles of its components: 1. Alkaloids and phenylpropanoids reduce capillary permeability by regulating Ca2 + concentration in vascular smooth muscle cells, achieving hemostasis. 2. Quinones and terpenes facilitate hemostasis by influencing platelet activation, adhesion, and deformation. 3. Flavonoids, organic acids, and glycosides promote the coagulation system by regulating prothrombin time, thrombin time, activated partial thromboplastin time, and activating coagulation factors. 4. Phenylpropanoids and steroids inhibit fibrinolysis by blocking fibrinolytic protein, fibrinogen, and fibrin binding, and by regulating fibrin production and fibrinase activity [17].

In traditional Chinese medicine, blood loss is categorized as "blood syndrome," where the vessel of blood is considered the house of blood. Under normal circumstances, blood in the vessel nourishes the entire body. When muscles and bones are injured, causing blood to overflow outside the vessel, it is termed “blood stasis.” Numerous scholars have confirmed that the Chinese herbal medicine prescription "Siwu decoction" can significantly reduce hidden blood loss after IFF [50, 51]. "Bazhen decoction" not only reduces hidden bleeding after IFF but also accelerates the recovery rate of haemoglobin and red cell volume. Liao et al. observed that Jiaoqi Yangxue Granules enhance physical fitness and hematopoietic function in mice with Qi and blood deficiency, promoting HB increase by elevating erythropoietin levels in the blood [59]. Liu et al. found that Siwu decoction improves the morphology and expression of red blood cells in chemo-induced anaemic mice, increases HB levels in peripheral blood, and enhances the expression of antigens on peripheral blood and spleen red blood cells, which may explain its mechanism in reducing hidden bleeding after IFF [60]. Some CHM formulations contain mineral drugs. Liu et al. discovered that mineral drugs, rich in Fe2+, Fe3+, Ca2+, Na+, and K+, are absorbed by the human body and interact with proteins or activate clotting factors via secondary messengers, promoting thrombin production and fibrin clot formation, thus exhibiting hemostatic effects [61]. However, according to the previous meta-analysis and systematic review on hidden blood loss, no researchers have analyzed CHM, and the advantages of tranexamic acid are obvious by simply comparing tranexamic acid with conventional drugs. Tranexamic acid significantly reduces hidden blood loss without increasing the risk of complications [62, 63]. With the prominent side effects of tranexamic acid, CHM may become an alternative medicine. Besides, current studies primarily compare the efficacy of a single type of CHM with conventional treatment, rather than examining the efficacy of different CHMs. It remains unclear which CHMs effectively reduce hidden blood loss and stabilize HB levels, which CHMs best reduce hidden blood loss, and which have fewer adverse reactions. The prescriptions that were particularly effective in this study largely fall into the categories of blood deficiency treatment and removing blood stasis. This study aims to explore these effects through network meta-analysis to provide clinical guidance.

This study investigated the effect of CHM on RCT performance in patients with IFF postoperative hidden bleeding through a web-based meta-analysis. A total of 31 studies, encompassing 17 CHMs, were included. Traditional meta-analysis revealed that, in terms of improving postoperative HB, conventional treatment combined with Danggui Buxue decoction, Sanqi powder, Siwu decoction, Guibi decoction, Shiquan Dabu decoction, Qitian Keli prescription, Taohong Siwu decoction, Yiqi Buxue decoction, Fuyuan decoction, and Yangxue Rougan decoction were superior to conventional treatment alone. Additionally, these CHM combinations were more effective in reducing postoperative hidden bleeding. Specifically, Bazhen decoction, Siwu decoction, Buyang Huanwu decoction, Guipi decoction, Shiquan Dabu decoction, Yangxue Rougan decoction, Qitian Keli prescription, Taohong Siwu decoction, and Yiqi Buxue decoction outperformed conventional treatment in this regard. For improving the Harris score, conventional treatment combined with Danggui Buxue decoction and Bazhen decoction significantly enhanced hip joint function compared to conventional treatment alone. Network meta-analysis indicated that, for postoperative HB improvement, the top three interventions were conventional treatment combined with Yiqi Buxue decoction, Yangxue Rougan decoction, and Sanqi powder. In terms of reducing hidden bleeding, the top interventions were conventional treatment combined with Siwu decoction, Yangxue Rougan decoction, and Qitian Keli prescription. Yangxue Rougan decoction demonstrated significant efficacy in both improving postoperative HB and reducing hidden blood loss, making it clinically recommendable. For improving the Harris score, the top interventions were conventional treatment combined with Bazhen decoction, Danggui Buxue decoction, and Sanqi powder. Both conventional and network meta-analyses indicated similar patterns, with some variances likely due to the limited number of studies, small sample sizes, and subject categorization based on syndrome differentiation.

In conclusion, compared to conventional treatment, CHM has shown advantages in improving postoperative HB, reducing hidden bleeding, and enhancing the Harris score, making it worthy of clinical recommendation.

In terms of safety, CHM combined with conventional treatment results in fewer adverse reactions compared to conventional treatment alone, indicating superior safety. Regarding anaphylaxis, only one case was reported in both the observation and control groups, suggesting a low incidence and acceptable safety profile for CHM. Digestive reactions, such as nausea and diarrhoea, were observed in 5 cases in the observation group and 3 cases in the control group. Although the observation group had a slightly higher incidence, the overall occurrence was low, warranting clinical attention. Deep vein thrombosis was reported in 6 cases in the observation group and 19 cases in the control group, with a higher incidence in the control group likely due to adverse reactions from anti-fibrinolytic drugs. The lower incidence in the observation group indicates that CHM not only effectively reduces hidden blood loss but also mitigates the risk of DVT. For hemorrhagic anaemia, the observation group exhibited a lower incidence compared to the control group, demonstrating the efficacy of CHM in preventing blood loss. Lung infections were less frequent in the observation group, possibly due to the anti-infective properties of CHM, although further research is needed to confirm this. Other adverse reactions, such as headaches, dizziness, incision complications, and systemic complications, showed minimal differences between the groups, requiring further verification in subsequent studies. One case of abnormal liver function was reported in the observation group, but none in the control group, highlighting the need for monitoring liver function during CHM use in clinical practice.

Although the cost of iron and functional exercise is lower than that of CHM, these options are not discussed here because iron does not fundamentally reduce hidden bleeding in IFFs, and functional exercise has minimal effect. Blood transfusion is widely used in developed countries; however, the cost of sourcing and maintaining blood supplies is prohibitively high and significantly exceeds the cost of CHM. Blood transfusion is also less feasible in developing countries. In terms of cost, the focus is on anti-fibrinolytic drugs. In China, the price of imported anti-fibrinolytics, such as tranexamic acid, is approximately 300 RMB per dose. Although the price drops to about 50 RMB with centralized drug procurement, a dose of CHM costs only 30 RMB. Furthermore, with government promotion and inclusion in the health insurance system, the cost of CHM can fall to 10 RMB per dose. Since CHM is not widely used outside China, global cost comparisons are challenging. However, plant-based medicines generally do not incur high costs. Thus, it can be concluded that CHM is cost-effective in many countries and regions. Compared to blood transfusion and anti-fibrinolytic drugs, CHM offers a balance of cost and efficacy that justifies its value.

## Limitations

This study has some limitations: (1) The inconsistency in the quality of the incorporated studies could affect the reliability of the findings. Among the 31 studies, 9 utilized high-risk random allocation methods, and only 2 out of 22 low-risk random allocation methods mentioned allocation concealment. Additionally, the other studies did not reference the use of blinding or the concealment of allocation. The GRADE results showed that the evidence quality of all studies was medium or low. (2) The exclusion of English literature from this research could potentially result in biased and selective reporting. (3) The varying durations of observation across the included studies may impact the accuracy of the results. (4) The inclusion criteria of some studies did not specify CHM syndrome types and lacked "syndrome differentiation and treatment," which compromised the reliability of the results. These limitations may contribute to the heterogeneity among studies. Future research should involve larger sample sizes and higher-quality RCTs to improve the verification process.

## Conclusions

Yangxue Rougan decoction not only effectively improves postoperative HB but also significantly reduces postoperative hidden blood loss, making it a preferred choice. Yiqi Buxue decoction is recommended for patients with low HB before IFF surgery due to its efficacy in improving HB postoperatively. Siwu decoction is highly effective in reducing postoperative hidden bleeding, likely due to its preventative action against postoperative bleeding following IFF. For patients facing high surgical difficulty and risk, Bazhen decoction is advisable. It excels in improving hip joint function post-surgery, enhancing postoperative HB, reducing hidden bleeding, and aiding functional recovery. However, given the limited number of CHM studies focused on hip joint function scores, this recommendation should be considered with caution. Regarding adverse reactions, CHM combined with conventional treatment exhibits fewer adverse reactions and good safety. Clinically, treatment approaches should be tailored to each patient's unique condition, with outcome rankings serving as a reference for medical professionals. Further rigorous RCTs are necessary to compare different CHM treatments to provide the best evidence for clinicians.

## Supplementary Information

Supplementary file 1 

## Data Availability

No datasets were generated or analysed during the current study.

## Abbreviations

CHM: Chinese herbal medicine
IFF: Intertrochanteric femur fracture
RCT: Randomised controlled trial
HB: Hemoglobin
MD: Mean difference
SMD: Standardised mean difference
CI: Confidence interval
SUCRA: Surface under the cumulative ranking curve
PRISMA: Preferred Reporting Items for Systematic Reviews and Meta-Analyses
CNKI: Chinese National Knowledge Infrastructure
VIP: China Science and Technology Journal Database
Wanfang: Wanfang Data Knowledge Service Platform
PROSPERO: Prospective Register of Systematic Reviews
