# Supplementary material for: Identifying an effective Chinese herbal medicine for reducing postoperative hidden bleeding and stabilizing blood volume following intertrochanteric femur fracture: a Bayesian network meta-analysis of randomized controlled trials
Source: J Orthop Surg Res. 2024 Dec 26;19:876. doi: 10.1186/s13018-024-05379-3 (PMC11670391; doi:10.1186/s13018-024-05379-3)
Supplement: Supplementary file 1 [file 13018_2024_5379_MOESM1_ESM.docx]

Pubmed:
(((Randomized Controlled Trial[Publication Type]) AND (Hemorrhage OR Hemorrhages OR Bleeding)) AND (Hip Fractures OR Fractures, Hip OR Intertrochanteric Fractures OR Fractures, Intertrochanteric OR Trochanteric Fractures OR Fractures, Trochanteric OR Trochlear Fractures, Femur OR Femur Trochlear Fracture OR Femur Trochlear Fractures OR Fracture, Femur Trochlear OR Fractures, Femur Trochlear OR Trochlear Fracture, Femur OR Femoral Trochlear Fractures OR Femoral Trochlear Fracture OR Fracture, Femoral Trochlear OR Fractures, Femoral Trochlear OR Trochlear Fracture, Femoral OR Trochlear Fractures, Femoral OR Subtrochanteric Fractures OR Fractures, Subtrochanteric)) AND (("Herbal Medicine"[Mesh] OR Herbal Medicine OR Medicine, Herbal OR Hawaiian Herbal Medicine OR Herbal Medicine, Hawaiian OR Medicine, Hawaiian Herbal OR La'au Lapa'au OR Laau Lapaau OR La au Lapa au OR Herbalism)

WOS

((TS=(Hemorrhage OR Hemorrhages OR Bleeding)) AND TS=(Hip Fractures OR Fractures, Hip OR Intertrochanteric Fractures OR Fractures, Intertrochanteric OR Trochanteric Fractures OR Fractures, Trochanteric OR Trochlear Fractures, Femur OR Femur Trochlear Fracture OR Femur Trochlear Fractures OR Fracture, Femur Trochlear OR Fractures, Femur Trochlear OR Trochlear Fracture, Femur OR Femoral Trochlear Fractures OR Femoral Trochlear Fracture OR Fracture, Femoral Trochlear OR Fractures, Femoral Trochlear OR Trochlear Fracture, Femoral OR Trochlear Fractures, Femoral OR Subtrochanteric Fractures OR Fractures, Subtrochanteric)) AND TS=(Herbal Medicine OR Herbal Medicine OR Medicine, Herbal OR Hawaiian Herbal Medicine OR Herbal Medicine, Hawaiian OR Medicine, Hawaiian Herbal OR La'au Lapa'au OR Laau Lapaau OR La au Lapa au OR Herbalism) and Preprint Citation Index

Cochrane library

(MeSH descriptor: [Hip Fractures] explode all trees) AND (MeSH descriptor: [Herbal Medicine] in all MeSH products) AND (MeSH descriptor: [Hemorrhage] explode all trees)

Chinese National Knowledge Infrastructure（CNKI）

(“股骨粗隆间骨折”+“股骨转子间骨折”)AND(“隐性失血”+“隐性出血”)AND(“汤”+“方”+“丸”+“粉”+“中药”)AND(“随机”+“随机对照”+“RCT”)，After advanced search and manual screening, 84 articles were finally obtained.

Wanfang Data Knowledge Service Platform （Wanfang）

(“股骨粗隆间骨折”OR“股骨转子间骨折”)AND(“隐性失血”OR“隐性出血”)AND(“汤”OR“方”OR“丸”OR“粉”OR“中药”)

，After advanced search and manual screening, 680 articles were finally obtained.

China Science and Technology Journal Database（VIP ）

(U=（股骨粗隆间骨折 OR 股骨转子间骨折）) AND (U=（隐性失血 OR 隐性出血）) AND (U=（汤 OR 方 OR 丸 OR 粉 OR 中药）)，After advanced search and manual screening, 89 articles were finally obtained.
